# Genetic fingerprinting and aflatoxin production of *Aspergillus* section Flavi associated with groundnut in eastern Ethiopia

**DOI:** 10.1186/s12866-021-02290-3

**Published:** 2021-08-28

**Authors:** Abdi Mohammed, Paola C. Faustinelli, Alemayehu Chala, Mashilla Dejene, Chemeda Fininsa, Amare Ayalew, Chris O. Ojiewo, David A. Hoisington, Victor S. Sobolev, Jaime Martínez-Castillo, Renee S. Arias

**Affiliations:** 1 School of Plant Science, College of Agriculture and Environmental Sciences, Haramaya University, P.O. Box 138, Dire Dawa, Ethiopia; 2 United States Department of Agriculture-Agricultural Research Service-National Peanut Research Laboratory, Dawson, GA 39842-0509 USA; 3 College of Agriculture, Hawassa University, P.O. Box 5, Hawassa, Ethiopia; 4 Partnership for Aflatoxin Control in Africa (PACA), African Union Commission, Addis Ababa, Ethiopia; 5 ICRISAT – Nairobi, UN-Avenue, Box 39063-00623, Nairobi, Kenya; 6 College of Agriculture and Environmental Sciences, Peanut and Mycotoxin Innovation Lab, University of Georgia, Athens, GA 30602-4356 USA; 7 Centro de Investigación Científica de Yucatán A.C., Unidad de Recursos Naturales, Calle 43 No. 130, Colonia Chuburná de Hidalgo CP 97200, Mérida, Mexico

**Keywords:** Aflatoxin, *Aspergillus*, Genetic diversity, Peanut, Insertion/deletion markers

## Abstract

**Background:**

*Aspergillus* species cause aflatoxin contamination in groundnut kernels, being a health threat in agricultural products and leading to commodity rejection by domestic and international markets. Presence of *Aspergillus flavus* and *A. parasiticus* colonizing groundnut in eastern Ethiopia, as well as presence of aflatoxins have been reported, though in this region, no genetic studies have been done of these species in relation to their aflatoxin production.

**Results:**

In this study, 145 *Aspergillus* isolates obtained from groundnut kernels in eastern Ethiopia were genetically fingerprinted using 23 Insertion/Deletion (InDel) markers within the aflatoxin-biosynthesis gene cluster (ABC), identifying 133 ABC genotypes. Eighty-four isolates were analyzed by Ultra-Performance Liquid Chromatography (UPLC) for in vitro aflatoxin production. Analysis of genetic distances based on the approximately 85 kb-ABC by Neighbor Joining (NJ), 3D-Principal Coordinate Analysis (3D-PCoA), and Structure software, clustered the isolates into three main groups as a gradient in their aflatoxin production. Group I, contained 98% *A. flavus*, including L- and non-producers of sclerotia (NPS), producers of B_1_ and B_2_ aflatoxins, and most of them collected from the lowland-dry Babile area. Group II was a genetic admixture population of *A. flavus* (NPS) and *A. flavus* S morphotype, both low producers of aflatoxins. Group III was primarily represented by *A. parasiticus* and *A. flavus* S morphotype isolates both producers of B_1_, B_2_ and G_1_, G_2_ aflatoxins, and originated from the regions of Darolabu and Gursum. The highest in vitro producer of aflatoxin B_1_ was *A. flavus* NPS N1436 (77.98 μg/mL), and the highest producer of aflatoxin G_1_ was *A. parasiticus* N1348 (50.33 μg/mL), these isolates were from Gursum and Darolabu, respectively.

**Conclusions:**

To the best of our knowledge, this is the first study that combined the use of InDel fingerprinting of the ABC and corresponding aflatoxin production capability to describe the genetic diversity of *Aspergillus* isolates from groundnut in eastern Ethiopia.

Three InDel markers, AFLC04, AFLC08 and AFLC19, accounted for the main assignment of individuals to the three Groups; their loci corresponded to *aflC* (*pksA*), *hypC*, and *aflW* (*moxY*) genes, respectively. Despite InDels within the ABC being often associated to loss of aflatoxin production, the vast InDel polymorphism observed in the *Aspergillus* isolates did not completely impaired their aflatoxin production in vitro.

**Supplementary Information:**

The online version contains supplementary material available at 10.1186/s12866-021-02290-3.

## Background

Groundnut (*A. hypogaea* L.) is an annual legume, important as a source of nutrition and income around the world. Ethiopia currently produces 78,475 MT of this crop [1], with the eastern parts of the country, mainly the East Hararghe region, accounting for 43% of groundnut production, where is replacing major crops in the area [2]. However, groundnut production and quality are hampered by the presence of *Aspergillus* fungi on kernels [3–5].

*Aspergillus* is a genus consisting of many species with worldwide distribution and adaptation to various climates [6]. Within the genus *Aspergillus*, section Flavi includes economically important species that can be divided into two main groups: the aflatoxigenic species (e.g. *A. flavus, A. parasiticus,* and *A. nomius*) and the non-aflatoxigenic species which include the domesticated (e.g. *A. oryzae* and *A. sojae*) [7] and the naturally occurring non-aflatoxigenic *A. flavus* strains [8–11]. The morphological complex species *A. flavus* [12] has two sclerotium-size morphotypes: the large (L)-morphotype, which produces few sclerotia that are > 400 μm in diameter and numerous conidiophores, and the small (S)-morphotype, which produces numerous sclerotia < 400 μm in diameter in association with few conidiophores [13]. Other isolates show typical *A. flavus* morphology though apparently have lost their ability to produce sclerotia in culture medium [12, 14, 15]. In the present work, isolates were grouped according to those general morphotypes, and those that did not produce sclerotia were referred as NPS.

Aflatoxins are mycotoxins produced primarily by species from *Aspergillus* section Flavi*,* such as *A. flavus, A. parasiticus,* and *A. nomius.* Many commodities used for human and animal consumption are contaminated with these toxins [16], which are known to be mutagenic, teratogenic, carcinogenic, and immunosuppressive [16, 17]. Fungi from section Flavi are able to produce: B_1_, B_2_, G_1_, and G_2_ aflatoxins, with type B_1_ being the most potent carcinogen known in nature [18]. Other aflatoxins such as M_1_ are naturally produced by *A. flavus* NRRL3251 [19], and other *A. flavus* isolates produce aflatoxins M_1_ and M_2_ [20].

Research has shown widespread presence of aflatoxins in groundnut and groundnut products in African countries [21, 22]. In eastern Ethiopia, reports of presence of *Aspergillus* spp. and aflatoxins in groundnut products has aimed to raise awareness of the risk such contamination poses to human health [4, 5, 23]. From hepatocellular carcinoma to growth impairment, aflatoxin contamination of food stuff is a constant threat [24, 25], and every few years aflatoxicosis results in human casualties [26].

Few technologies and methods are available to prevent the impact of aflatoxin contamination [27, 28] but these are often not affordable in developing countries. One strategy is the application of atoxigenic strains that can out-compete the toxigenic ones. For example, the product Afla-guard®, that contains the atoxigenic strain of *A. flavus* NRRL 21882, was able to reduce 88% of aflatoxin contamination in peanut fields [29]. However, not all geographic areas are colonized by the same strains of *Aspergillus*, for example, West African *A. flavus* S morphotype isolates differed from North American isolates in aflatoxin type and quantity produced [30]. Therefore, biocontrol programs with *Aspergillus* require understanding the population biology of this fungus in the region of interest before the control is implemented [31]. The most successful results in aflatoxin control have been accomplished by using native non-aflatoxin-producing strains [32]. One of the objectives of the present work was to identify the most common *Aspergillus* genotypes colonizing groundnut in Ethiopia by using ABC InDels to later subject them to whole-genome sequencing. Such information could be used in designing targets for RNA-interference-mediated gene silencing of aflatoxin synthesis genes that could be potentially effective against the most common genotypes. A secondary objective was to find non-aflatoxigenic isolates that could be used in biological control in the region. The workflow used in the present work performing InDel fingerprinting of the ABC of isolates has demonstrated this approach gives comparable results as cluster analysis of the complete ABC genomic sequences (~ 100 kb) of selected isolates [11].

Twenty-five genes are involved in aflatoxin biosynthesis in *Aspergillus* [33] and genetic diversity exists within the cluster among species [11, 34]. InDels within the ABC influence aflatoxin biosynthesis [35] which makes InDel marker a valuable tool for characterizing intraspecific variations [11]. InDel markers have been used to monitor non-aflatoxigenic *A. flavus* strains [36] and to distinguish groups according to their aflatoxin profile and genotype abundance in a geographic region [11].

There is a knowledge gap in the genetic diversity and aflatoxin production capacity of *Aspergillus* species in Ethiopia, which is hindering efforts to select non-aflatoxigenic *Aspergillus* isolates as potential biocontrol agents. In the current study, InDels were used to analyze the genetic diversity of *Aspergillus* species isolated from groundnut samples in eastern Ethiopia, and the production of aflatoxins was determined for selected isolates. Potential associations between fungal genotype and geographic area, as well as identification of the most frequent genotypes of *Aspergillus* in the groundnut-producing area of eastern Ethiopia are described.

## Results

### Genetic diversity of *Aspergillus* isolates

A total of 145 isolates of *Aspergillus* obtained from groundnut kernels collected during the 2014/15 season were evaluated (Supplementary Table 1); and the methodology for isolation and identification of these isolates has already been published [23]. Additional morphological observations of the isolates, such as colony color, conidia and sclerotium size indicated that *A. flavus* NPS was the predominant species (69%, *n* = 101) followed by *A. parasiticus* (15%, *n* = 22) and *A. flavus* S-morphotype (14%, *n* = 21), while *A. flavus* L-morphotype was the least abundant (1%, *n* = 2). *A. flavus* S- and L- morphotypes were distinguished by presence of sclerotia smaller or larger than 400 μm, respectively.

**Fig. 1:**
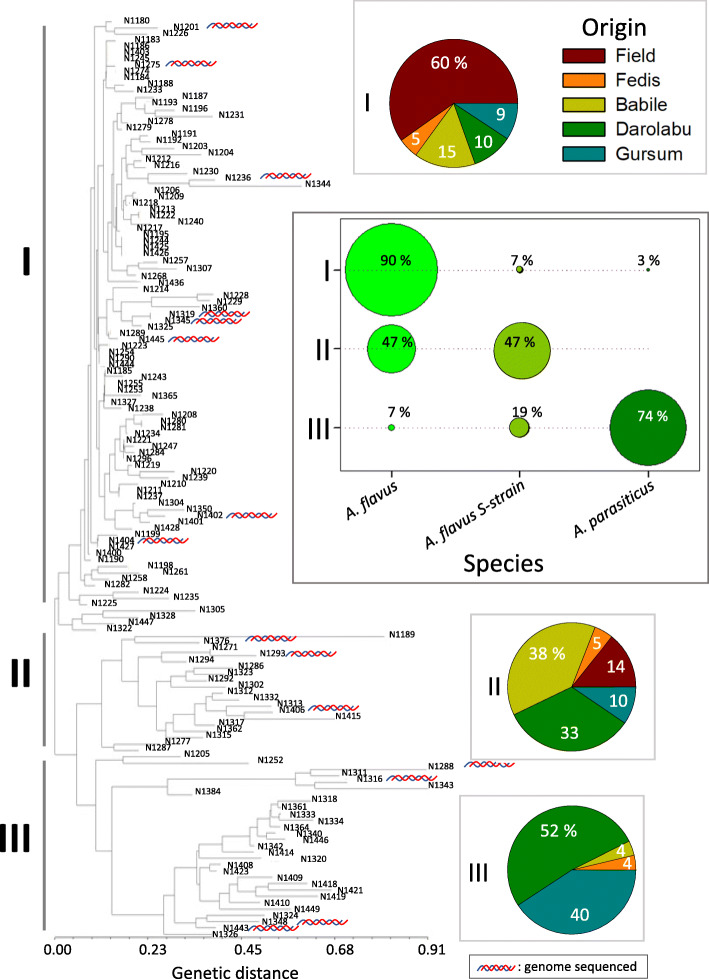
Neighbor Joining (NJ) analysis of the genetic distances estimated from the aflatoxin biosynthesis cluster. NJ of 145 *Aspergillus* isolates from groundnut kernels in Ethiopia based of genetic fingerprinting of 23 InDels in the aflatoxin-biosynthesis pathway. Three main groups were identified: **Group I** consisted mainly of *A. flavus* sclerotia-non producers (NPS). **Group II** included mainly *A. flavus* S-strain and *A. flavus* NPS, all producers of type B aflatoxin. **Group III** comprised primarily *A. parasiticus* with a small sub-clade of *A. flavus* S-strains, all producers of B and G aflatoxins. Symbol : indicates isolates from which their genomes have been sequenced (Arias et al. 2020). Percentage of each of the species present within each group is indicated in a separate frame

A total of 23 InDel markers utilized to assess the isolates for genetic variations within the ABC identified 133 different genotypes (Fig. 1), the DNA sequences of 22 of these markers had been published [11]. Most markers amplified all the isolates, but two markers, AFLC14 and AFLC25, did not amplify 31 and 26% of the isolates, respectively. Since the amount of DNA was not a limiting factor for other markers to detect amplicons, the lack of amplification in these two markers was considered as presence of null alleles. InDel markers detected a total of 123 amplicons, these ranged from 2 to 11 per marker, with an average of 5 (Table 1). All raw data of fingerprinting have been deposited at Harvard Dataverse repository, with persistent weblink: 10.7910/DVN/CXX0TG.

**Table 1:** InDel markers within the aflatoxin biosynthesis cluster used for fingerprinting *Aspergillus* species

Marker name	Relative position	Forward 5′ → 3′	Reverse 5′ → 3′	Amplicon size range (bp)	Amplicons/sample (mean ± SE)	Number of amplicons	Fingerprint reference to raw data
AFLC01	190–340	CCGACCTCACGACGCATTAT	CCGGCTAGCTTCAACAGACG	127–370	0.80 ± 0.36	6	*AFLC01*
AFLC02	1367–1521	GGTTGGCGGATTGAGAGGTA	GGAGATCAGCCGAGAAGACA	161–216	1.00 ± 0.00	6	*AFLC02 – AFLC14*
AFLC03	5738–5868	TCCGCCGAGAGCCATAATAG	GGATGCTGACACCTCGATAG	152–155	1.00 ± 0.00	3	*AFLC15*
AFLC04	7897–8073	ACAGCTGGCATGCTCCGTAT	ATTGCTGCGCACGACGCTTA	194–199	1.00 ± 0.00	4	*AFLC16*
AFLC05	11633–11783	GTGGATGGACTGCCACTTAG	AGACCACAGTGAGTGCTTCT	161–195	1.00 ± 0.00	9	*AFLC17*
AFLC06	12333–12543	GCTGTCCTGGACGGATAGTA	CATCGGTCAACGACGAAGTA	230–232	1.00 ± 0.00	3	*AFLC18*
AFLC07	12715–12889	GTCAGCAAGAGGAGCCTTCA	GGTCACGGAGATCCTCCATA	160–197	1.00 ± 0.00	3	*AFLC03*
AFLC08	14001–14235	CGCCAGCACGGAGATCGAAT	CGTCTCCTCAGGCGGTCTAT	243–257	1.00 ± 0.00	5	*AFLC20*
AFLC09	16162–16331	AACACTCCGCTGCTCAACTA	AACGCTCAGGCAACGTCGAA	131–318	1.00 ± 0.00	7	*AFLC04*
AFLC10	16315–16498	GACGTTGCCTGAGCGTTAAT	TGACTGGTCGTCGCCAGAAT	135–218	1.00 ± 0.08	9	*AFLC21*
AFLC11	21671–21862	CTCGACGTAGCGTTGAACAG	AACGCATGGCCAGCTAATCT	157–228	1.00 ± 0.11	5	*AFLC22*
AFLC12	21895–22149	CGCAAGGAGCTCGACCAATA	TTCAGCTCAGCGACGAGAGT	134–278	1.00 ± 0.19	6	*AFLC23*
AFLC13	22059–22241	TCGGTTCAATGCTCGAACAC	TCCAACCTTCGGCCTAGTCT	183–194	1.00 ± 0.08	3	*AFLC24*
AFLC14	22155–22242	GACGCCTCGGCTTGTCAAGA	CTCCAACCTTCGGCCTAGTC	96–121	0.80 ± 0.44	3	*AFLC05*
AFLC18	62617–62825	GGCAGCCAGACCAAGGAATA	CCTTCTCGTAGCCGCTCATC	230–231	1.00 ± 0.00	2	*AFLC13*
AFLC19	63261–63509	ACAGGACCGCACGGATCAAT	AGGAGCGGATGTCGAAGTCT	260–270	1.00 ± 0.00	6	*AFLC12*
AFLC20	67451–67735	GCCTAGCGCTCCATTCTCAG	CCATCGTATCCGGCTCTATC	262–369	1.00 ± 0.11	11	*AFLC11*
AFLC21	68690–68852	TACCTTACTCCGCTAAGCAG	GCGGTCACCTACCAATGAAT	169–321	1.00 ± 0.19	9	*AFLC10*
AFLC22	68718–68959	TTCGCAGGAGTGTAGCCAAG	GTTGGAACACGCTCCATAGG	259–269	1.00 ± 0.11	7	*AFLC09*
AFLC23	72035–72162	GGCGTCAGTGGATTCCGGAT	CGTGGTCCGCAGCAATAGTG	140–156	1.00 ± 0.11	3	*AFLC08*
AFLC24	73119–73357	GAACGAGATAACGGCTGCAT	ATCAATCCACGGACCGTTGT	260–261	1.00 ± 0.19	2	*AFLC06*
AFLC25	72652–72811	CAGTGCGACCGGATGGTACA	CGGCTGAACGCGATGACTCT	110–184	0.80 ± 0.39	5	*AFLC07*
AFLC26^a^	13627–13702	CGGCGTGGTGCGGTACTAAT	TAATACGCGCCGGCATCTCC	89–95	1.00 ± 0.00	6	*AFLC19*

*SE* standard error of the mean

^a^Marker used only in this study, while the rest adopted from Faustinelli et al. [11]. Marker names in italics, correspond to the labels used in the raw data files of fingerprinting DOI: 10.15482/USDA.ADC/1520771

NJ analysis [37] distinguished three main groups of isolates, labelled as I, II, and III in Fig. 1. Group I (*n* = 97) was the largest, comprising mainly *A. flavus* (97%), most of them (91%) NPS, and few (7%) S-morphotype isolates. Group II (*n* = 21) had a similar number of *A. flavus* NPS (47%) and *A. flavus* S-morphotype (47%). Group III (*n* = 27) included mostly *A. parasiticus* (74%), followed by *A. flavus* S-morphotype (19%), and a small percentage of *A. flavus* NPS (7%), Fig. 1. The geographic origin of most isolates in Groups I and II, 75 and 52%, respectively, was mainly from the low-land dry area of Babile, which includes samples labelled Field and Babile. Whereas isolates in Group III were mainly from Darolabu (52%) and Gursum (40%), Fig. 1.

**Fig. 2:**
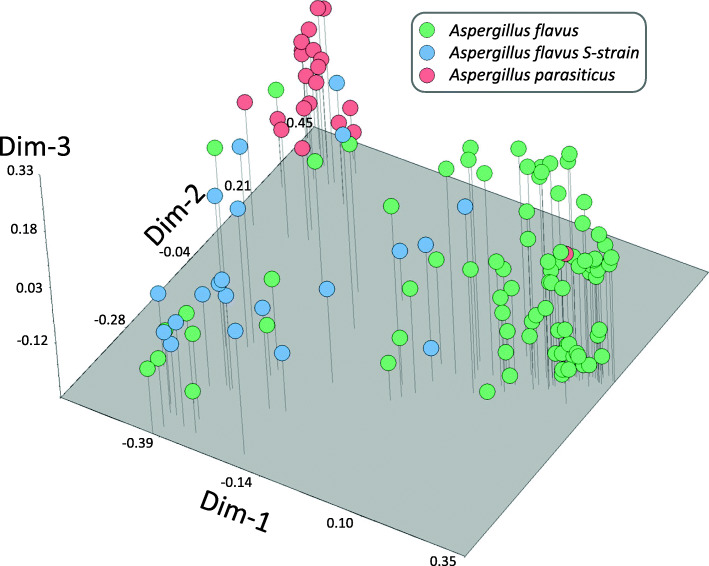
3D-Principal Coordinate Analysis (3D-PCoA) of groundnut *Aspergillus* isolates. 3D-PCoA of 145 *Aspergillus* isolates from groundnut from eastern Ethiopia using 23 InDel markers. The first coordinate (Dim-1) distinguished most of the *A. flavus* that belong to **Group I**. The second coordinate (Dim-2) contributed to the differentiation of **Group II** (most of them *A. flavus* S-strain) and **Group III** containing mainly *A. parasiticus* isolates

A 3-D Principal Coordinate Analysis (3D-PCoA) was evaluated from binary data of DNA fingerprinting used in Neighbor Joining analysis, Fig. 2. The 3D-PCoA categorized by species showed that the first three coordinates explained 88% of the total observed variation. The first coordinate (Dim-1; 53%) clustered together most of the *A. flavus* that belong to Group I in NJ analysis; whereas the second coordinate (Dim-2; 22%) discriminated Group II (mostly *A. flavus* S-morphotype) from Group III (mostly *A. parasiticus*), Fig. 2.

Structure analysis was done for different values of parameter K. The K value that captured the major structure in our data was K = 2 (ΔK = 1231.9) indicating that based on the data of ABC fingerprinting there were only two major genetic groups, *A. flavus* and *A. parasiticus*. The *A. flavus* group was formed mainly by isolates from the Babile area, whereas the *A. parasiticus* group contained isolates mainly from Darolabu and Gursum.

**Fig. 3:**
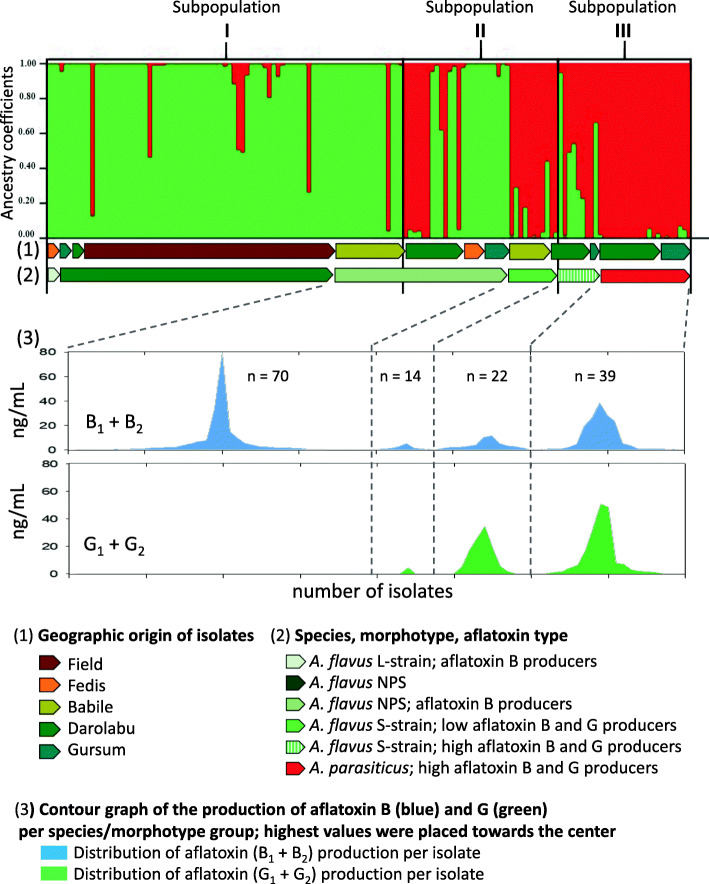
Population Structure Analysis of groundnut *Aspergillus* isolates. Population structure of 145 isolates from eastern Ethiopia evaluated using genetic fingerprinting of 23 InDels within the aflatoxin-biosynthesis gene cluster. K = 2; ΔK = 1231.9

In our study, the detected partition consisted of three Groups. **Group I** contained most of the *A. flavus*-NPS that produced aflatoxins B_1_ and B_2_ (henceforth referred as type B) and geographically originated from the Babile area (Field + Babile), Fig. 3. **Group II** was a transitional admixture group, containing *A. flavus* NPS producing only low levels of aflatoxin B, and S-morphotype producing low levels of type B aflatoxins as well as aflatoxins G_1_ and G_2_ (henceforth referred as type G); this Group comprised isolates from all the areas tested. **Group III** included mostly *A. parasiticus* followed by *A. flavus* S-morphotype, both groups being producers of aflatoxin type B and G, and mostly originated from the highland-humid areas of Darolabu and Gursum, Fig. 3. NJ groups I and III had 79 and 89% of the same isolates as Group I and III from the Structure analysis, respectively.

Some common features were found within Groups by DNA fingerprinting of InDels using capillary electrophoresis. For example, for marker AFLC19, most of the isolates in Group I presented a 2 bp deletion when compared to Group II, or 3 bp deletion if compared to Group III. Marker AFLC19 is located within the *aflW* (*moxY*) gene, and only few samples showed null alleles for this locus. In Group II, comprised by *A. flavus* NPS aflatoxin-B producers and *A. flavus* S-morphotype, both producing almost exclusively B-aflatoxins, most of the individuals were distinguished from other Groups by a single allele in marker AFLC08. The polymorphic locus of AFLC08 is in the intergenic region between the *hypC* and *aflD* genes. Finally, all isolates from Group III were distinguished from the other Groups by a single allele in marker AFLC04 with locus on the *pksA* gene which encodes a polyketide synthase at the beginning of the aflatoxin biosynthesis pathway.

### In vitro aflatoxin production of *Aspergillus* isolates

Aflatoxins B_1_, B_2_, G_1_, and G_2_ were screened in 84 isolates obtained from groundnut kernels. Average aflatoxin production by each type of isolates is shown in Table 2, and the complete list of results are shown in Additional file 1. The highest aflatoxin B_1_ producer (*A. flavus* NPS N1436 from Gursum; 77.98 μg/mL), and the highest aflatoxin G_1_ producer (*A. parasiticus* N1348 from Darolabu; 50.33 μg/mL) were found within Groups I and III, respectively. The 62 isolates from Field experiments were not evaluated for aflatoxin production as these came from the same Babile location from where many other samples had already been analyzed for aflatoxin production and had shown genetic similarity by clustering together both in Neighbor Joining as in Structure analyses.

**Table 2:** Average aflatoxins produced by isolates

	AFLATOXINS (μg/mL)			
ISOLATES	B_1_	B_2_	G_1_	G_2_
	Mean ± SE	Mean ± SE	Mean ± SE	Mean ± SE
*A. flavus* L-strain (*n* = 2)	**14.16 ± 11.12**	0.21 ± 0.17	0.00 ± 0.00	0.00 ± 0.00
*A. flavus* NPS (*n* = 39)	**4.94 ± 2.16**	0.10 ± 0.05	0.00 ± 0.00	0.00 ± 0.00
*A. parasiticus* (*n* = 22)	**7.26 ± 2.42**	0.26 ± 0.08	**8.74 ± 3.27**	0.12 ± 0.05
*A. flavus* S-strain (*n* = 21)	2.89 ± 0.64	0.09 ± 0.02	**12.5 ± 2.53**	0.13 ± 0.03

High values highlighted in bold

*n* number of individuals, *SE* standard error of the mean

## Discussion

This is the first study that characterized the genetic diversity of the ABC of Aspergilli section-Flavi by combining InDel fingerprinting of their ABC and the corresponding aflatoxin production of isolates collected from groundnuts in eastern Ethiopia. Neighbor Joining and 3D-PCoA analyses based on DNA fingerprinting of 23 InDels within the ABC separated Ethiopian isolates in three groups: Group I, *A. flavus* which produced type B aflatoxin, Group II, an admix of *A. flavus* and *A. flavus* S-morphotype, both producers of low levels of aflatoxins, and Group III, *A. parasiticus* with a subclade of *A. flavus* S-morphotype, a group in which most isolates were high producers of G and B aflatoxins, Figs. 1 and 3. S-morphotype is known to produce both main types of aflatoxins, B and G [30, 38, 39]. Using the same InDels, genetic fingerprinting of *Aspergillus* isolates from groundnuts in Georgia, USA, also identified three main groups, though in that study, one group of isolates did not produce aflatoxins, and no S-morphotype were observed [11].

Even though Structure analysis of the Ethiopian isolates based on ABC InDels clearly distinguished two major genetic groups, corresponding mainly to *A. flavus* and *A. parasiticus*, a third group was observed using PCoA and NJ which are more robust to missing genotype data [40], positioned *A. flavus* S-morphotype as a transition group between those groups, Fig. 3. We estimated the ancestral components of 145 isolates of *Aspergillus* section Flavi from Ethiopia peanut kernels, using Structure program (K = 2) and 23 InDel markers. Group I to III represent groups with different ancestral components. Researchers working with RAPD data and the sequence of an amylase gene, had also placed *A. flavus* S-morphotype as phylogenetically intermediate between *A. flavus* and *A. parasiticus* [41]; more recently, several new species names have been proposed within the S-morphotype group [42–44]. The goal of the present work was to determine variations within the ABC and identify predominant genotypes in relation to their aflatoxin production; thus, no taxonomic identification of the isolates was done at the molecular level. Despite that InDels within the ABC in *Aspergillus flavus* are in some instances associated with loss of aflatoxin production [35], all Ethiopian *Aspergillus* section Flavi isolates produced detectable levels of aflatoxins in vitro. InDels have been used to monitor non-aflatoxigenic *Aspergillus* spp. [36] and to characterize the genetic diversity of *Aspergillus* spp. to later sequence the genomes of the most frequent genotypes [11, 45]. In a similar approach, the genomes of 16 of the 145 Ethiopian *Aspergillus* isolates presented here have already been sequenced [46], as indicated in Fig. 1.

Group I through III also showed a gradient in chemotype, from producers of aflatoxin B in Group I, to producers of high levels of B and G aflatoxins in Group III, Fig. 3. Since all the isolates tested produced aflatoxins, no group of isolates corresponded to the aflatoxin non-producers (clade IB-type or GI) that had been described in the literature [11, 47], respectively.

Three InDels, AFLC19, AFLC08 and AFLC04, mainly accounted for the assignment of isolates to Groups I, II and III, respectively. In Group I, most of the isolates had a 3 bp insertion in the *aflW* (*moxY*) gene detected by marker AFLC19. This group consisted mostly of *A. flavus* NPS and few *A. flavus* L-morphotype isolates. The monoxygenase encoded by *aflW* [48] catalyzes the reactions HVN (hydroxyversicolorone) to VHA (versiconal hemiacetal acetate) and VONE (versicolorone) to VOAc (versiconol acetate), both oxidative steps required for aflatoxin biosynthesis [49]. In our study, the insertion found in the *aflW* gene of Group I isolates did not result in loss of aflatoxin production.

Isolates within Group II shared similar amplicons for InDel AFLC08, showing a 14 bp deletion in the anthrone-oxidase gene *hypC*, a gene in the intergenic region between *aflC* (*pksA*) and *aflD* (*nor-1*). The monooxygenase coded by *hypC* converts norsolorinic acid anthrone to norsolorinic acid, a precursor of aflatoxins [50]. The deletion in *hypC* resulted in the grouping of *A. flavus* NPS aflatoxin-B producers together with *A. flavus* S-morphotype that were low producers of aflatoxins B and G.

Group III comprised primarily *A. parasiticus* but also included a subclade of *A. flavus* S-morphotype capable of producing aflatoxin types B and G. Whereas production of type B and G aflatoxins is characteristic of *A. parasiticus*, it is also known that *A. flavus* S-morphotype can produce B and G aflatoxins [47]. Group III was distinguished from the rest by a 3 bp deletion in the *pksA* gene detected by marker AFLC04; this gene is required in the early steps of aflatoxin biosynthesis [51]. Search of the AFLC04 locus in NCBI showed that *A. parasiticus* CP051029.1, AY371490.1, L42765.1, Z47198.1, ML734987.1, JZEE01000728.1, LOAP01000469.1 did not have the mentioned 3 bp deletion. However, *A. parasiticus* isolates E1319, E1348, E1443 and E1337 [46] had a 3 bp deletion within the AFLC04 locus, being in E1337 slightly downstream. Despite that in some cases a single nucleotide polymorphism in the *pksA* gene has caused premature termination of protein synthesis and resulted in no aflatoxin production [52], in the Ethiopian isolates of *Aspergillus*, the 3 bp deletion did not completely impaired aflatoxin-producing capability.

The percentage of *Aspergillus* section Flavi isolated from groundnuts and able to produce aflatoxins can vary, for example in Vietnam 38% of the isolates were aflatoxigenic [31] whereas in the USA 96% of the isolates produced aflatoxins [11], in the present study, 100% of *Aspergillus* isolates produced detectable levels of aflatoxins in vitro. Various authors have described the prevalence of aflatoxin contamination in groundnut in Ethiopia [3, 4, 23]; however, no referable data are available on the aflatoxin producing potential of individual isolates. Using the same methods for isolation and UPLC aflatoxin analysis applied in the present work, we had found that 3.3% of the *Aspergillus* isolates from groundnut did not produce aflatoxins [11], thus we expected to find at least two aflatoxin-non producers among the isolates. No isolate has been identified in the present work as a non-producer of aflatoxins and with potential use in biocontrol.

In the present work, some association was observed between *Aspergillus* genotypes and their geographic origin; for example, most of the isolates from Babile were *A. flavus* producers of type B aflatoxins; whereas those from Gursum and Darolabu were mostly *A. parasiticus* and *A. flavus* S-morphotype, both producers of B and G aflatoxins. While Babile is a lowland area with 1590 m average altitude and < 900 mm mean annual rainfall, the areas of Darolabu and Gursum are highland areas at 1720 m altitude and > 1000 mm rainfall [5]. If we consider a dry adiabatic lapse rate of − 1 °C every 100 m-increase in altitude [53], the areas of Gursum and Darolabu would be an average of 1.3 °C cooler than Babile. Studies over a range of water activities have shown that *A. parasiticus* grows better at marginally cooler temperatures, approximately 3 °C lower [54], than *A. flavus* [55]. Since environmental factors such as drought and elevated soil temperatures are important factors determining the severity of groundnut kernel colonization by *Aspergillus* species [56, 57], then, it is possible that the slightly higher altitude in Darolabu and Gursum may have favored groundnut colonization by *A. parasiticus*. Alternatively, groundnut plants grown at higher altitude possibly provide better conditions for the colonization by *A. parasiticus*. Further work will be necessary to determine whether this is an actual trend.

## Conclusions

This is the first study of the genetic diversity of *Aspergillus flavus* and *A. parasiticus* isolates that colonize groundnut kernels in the main crop areas of Ethiopia, work performed using InDel loci within the ABC. Three groups were found, and these were mainly discriminated by InDels on three loci, aflW (moxY), aflC (pksA) and *hypC*. Despite the genetic polymorphism observed within the ABC, all isolates tested produced aflatoxins. Determining the most abundant species and genotypes colonizing groundnut in a particular area can provide basic information to develop new technologies for the control of aflatoxins.

## Methods

### Origin of *Aspergillus* isolates

During the 2014/2015 growing season, 20 cultivated groundnut samples from each of four Ethiopian districts (Babile, Darolabu, Fedis, and Gursum) were obtained in addition to 60 cultivated groundnut samples harvested from a field experiment (labelled: Field) from the Babile district in accordance to local legislation. Peanut samples were exported from Ethiopia under Phytosanitary Certificate: No. 339283, issued by the Ministry of Agriculture Animal and Plant Health Regulatory Directorate, Addis Ababa, Ethiopia, on 07-31-2014; and brought to the United States under Animal and Plant Health Inspection Service (APHIS), Plant Protection and Quarantine (PPQ) permit Number P526–13-03711, the kernels were received at the National Peanut Research Laboratory for fungal isolation. From the groundnut samples 145 *Aspergillus* spp. of section Flavi were isolated, the methods of isolation and identification of the isolates have already been published [22].

### Genetic diversity of *Aspergillus* species

DNA was extracted from spores and/or sclerotia of *Aspergillus* isolates that had been grown for 5–10 days on Petri plates containing Modified Dichloran Rose Bengal (MDRB) medium [58]. The spores and/or sclerotia were harvested using sterile plastic loops (Fisher Scientific, Waltham, MA) and placed in 2 mL screw-cap tubes (Omni International, Kennesaw, GA) that contained 2 metal (2.4 mm ∅) and 2 zirconium (2.8 mm ∅) beads. Grinding was performed with an Omni Bead Ruptor (Omni International) at 5 m/s for 40 s. DNA was extracted using the DNeasy Plant Mini Kit in a QIAcube robot (both from Qiagen, Valencia, CA) according to manufacturer’s instructions for plant DNA extraction, and then quantified using Nanodrop 2000c spectrophotometer (Nanodrop Technologies Thermo Fisher Scientific, Waltham, MA).

### PCR amplification, fingerprinting and phylogenetic structures of *Aspergillus* isolates

DNA fingerprinting was done for 145 *Aspergillus* isolates using 23 InDel markers (Table 1) to analyze their genetic diversity, as previously described [11]. Forward InDel primers were 5′-tailed with the sequence 5′-CAGTTTTCCCAGTCACGAC-3′ to permit product labeling, and reverse primers were tailed at the 5′ end with the sequence 5′-GTTT-3′ to promote non-template adenylation [59]. Primer 5′-CAGTTTTCCCAGTCACGAC-3′ labeled with 6-carboxy-fluorescein (FAM) (IDT, Coralville, IA) was used for labelling the amplification of 10 ng DNA using Titanium Taq DNA Polymerase (Clontech, Mountain View, CA) in 5 μL reactions on an M & J thermal cycler (BioRad, Hercules, CA) at 95 °C for 60 s, 60 °C for 60 s (2 cycles), 95 °C for 30 s, 60 °C for 30 s, 68 °C for 30 s (27 cycles) and a final extension at 68 °C for 4 min. Fluorescently-labelled PCR fragments were analyzed on an ABI 3730XL DNA analyzer, and data were processed using GeneMapper 4.0 (both from Applied Biosystems, Waltham, MA).

Amplicons within the range of the fluorescent ladder reference, 100 and 500 bp in length, were used in the analysis. Presence or absence of amplicons was converted to a binary matrix and used to calculate genetic distances among isolates. Cluster analysis of InDels was performed by NJ [60] using NTSYSpc2.2 (Applied Biostatistics Inc.) [61]. The same data were used in 3D-Principal Coordinate Analysis (3D-PCoA) [62] and run with Jaccard’s distances in NTSYSpc2.2 [61]. Analysis of the population structure was performed with Structure 2.1 [63]. Since only two species of *Aspergillus* were observed among the isolates, though up to four groups could be expected given that S-morphotype and L-morphotype were present. L-morphotype corresponds to isolates that have the morphology of a typical *A. flavus* but have apparently lost their capability to produce sclerotia, and have been normally reported separately in the literature [12, 15]. We use a maximum K = 5 in case that some other species was present. The use of a K value higher than the four predicted could help detect a potentially cryptic genetic structure in the database generated by fingerprinting. The admixture model was used with correlated allele frequencies, 200,000 as a period of burn-in, and 500,000 iterations after burn-in to allow the Markov chain to reach stationarity. Ten independent simulations were run for each value of K, ranging from K = 1 to K = 5, checking for consistency across outputs. To obtain the appropriate K from the data according to Evanno et al. [64], we used the Structure Harvester program [65].

### In vitro aflatoxin production of *Aspergillus* isolates

A total of 84 *Aspergillus* isolates were screened for aflatoxin production. The other 61 isolates labelled as “Field”, originated from a 270 m^2^ area that had already been sampled and labelled as “Babile”, so no additional phenotypes were expected regarding aflatoxin production of “Field” isolates. The fungi were grown on yeast extract sucrose (YES) liquid medium, consisting of 150 g/L sucrose, 20 g/L yeast extract and 10 g/L soytone [66]. Four mL vials containing 2 mL of YES medium were inoculated with spores of *A. flavus* including L- and S-morphotype, and *A. parasiticus* using a sterilized needle. Inoculated vials with loose caps were incubated at 30 °C for 5 days. For extraction of aflatoxins, 1 mL of chloroform was added to each vial and vortexed for 10 s, then the vials were placed in the dark at room temperature for 24 h. After that, 300 μL of the chloroform layer were transferred to 4 mL vials and placed in a heated block at 45 °C. The solvent was removed by a stream of N_2_. The dry residue was re-dissolved in 500 μL methanol-water mixture (8:2, v/v), vortexed for 3 s, followed by application of 500 μL of acetonitrile and vortexed for an extra 3 s, then 500 μL of the mixture were applied to an Alltech 1.5 mL Extract-Clean minicolumn containing 200 mg basic aluminum oxide [67]. Aflatoxins were eluted by gravity and collected into a 700 μL UPLC vial (p/n: 186005221, Waters Co., Milford, MA). An aliquot of purified extract (1 μL) was injected into the UPLC system. Aflatoxin detection and quantification was performed using an Acquity UPLC instrument equipped with a Quaternary Solvent manager, a Sample Manager-FTN, a fluorescent detector-FLR, and an Acquity UPLC BEH C18 1.7 μm 2.1 × 50 mm analytical column (Waters Co., Milford, MA). The column temperature was 40 °C. The mobile phase was composed of water-methanol-acetonitrile mixture (70:20:10, v/v/v, respectively) and the flow rate was 0.25 mL/min. Analysis and data processing were performed with Waters Empower3 Chromatography Data Software (Waters Co., Milford, MA). Concentrations of aflatoxins were calculated as μg/mL by reference to the calibration curves obtained by injecting different amounts of corresponding commercial standards (Sigma, St. Louis, MO). The quantification limits of aflatoxins B_1_ and G_1_ were 0.100 μg/mL, and 0.015 μg/mL for aflatoxin B_2_ and G_2_, respectively. Working solutions of aflatoxins were prepared according to the protocol published by Sobolev and Dorner [67].

## Supplementary Information

**Additional file 1: Supplementary Table 1.***Aspergillus* isolates used for genetic diversity (*N* = 145) from Ethiopia of which, 84 isolates were screened for aflatoxin B_1_, B_2_, G_1_ and G_2_ production using UPLC protocol (μg/mL). nd = not detected; nt = not tested.

## Data Availability

The datasets generated and analyzed during the current study are available in the Harvard Dataverse repository. The raw data of the genetic fingerprinting as output of ABI 3730 sequencer, have been deposited at Harvard Dataverse repository, with persistent web link: 10.7910/DVN/CXX0TG. Complete data set of aflatoxin determinations is provided in Additional File 1. Fungal isolates are stored at the National Peanut Research Laboratory collection, special APHIS permits are required to work with these fungi.

## Abbreviations

InDel: Insertion/deletion
UPLC: Ultra-performance liquid chromatography
3D-PCoA: 3-Dimentional Principal Coordinate Analysis
NJ: Neighbor Joining
NPS: Non-producers of sclerotia
L-strain: *Aspergillus flavus* that produce large, > 400 μm diam sclerotia
S-strain: *Aspergillus flavus* that produce small, < 400 μm diam sclerotia
bp: Base pairs
ABC: Aflatoxin-biosynthesis gene cluster
